# Diagnostic utility of antigen detection rapid diagnostic tests for Covid-19: a systematic review and meta-analysis

**DOI:** 10.1186/s13000-022-01215-6

**Published:** 2022-04-13

**Authors:** Somaye Ghasemi, Narges Nazari Harmooshi, Fakher Rahim

**Affiliations:** 1 Cellular & Molecular Research Center, Medical Basic Sciences Research Institute, Ahvaz Jundishapur University of Medical Sciences, Ahvaz, Iran; 2 Epidemiology Deputy of Health, Health Centre, Ahvaz Jundishapur University of Medical Sciences, Ahvaz, Iran; 3 Phd Student Candidate Health in Emergency and Disaster Research Center, University of Social Welfare and Rehabilitation Sciences, Tehran, Iran; 4 Thalassemia and Hemoglobinopathy Research Centre, Ahvaz Jundishapur University of Medical Sciences, Ahvaz, Iran

## Abstract

**Background:**

The early detection of coronavirus disease (COVID-19) infection to improve disease management becomes the greatest challenge. Despite the high sensitivity of RT-PCR, not only it was reported that 20–67% of infected patients had false-negative results. Rapid diagnostic tests (RDTs) are widely used as a point-of-care test for SARS-CoV-2 detection in pharyngeal and blood specimens. It’s more appealing since it’s less time-consuming, doesn’t seem to be as expensive, and doesn’t need any specific training, but the poor sensitivity is the major limitation. Several reports indicated the rapid test of blood and pharyngeal samples has the same sensitivity as the RT-PCR, but some reports have lower sensitivity, especially in asymptomatic patients.

**Methods:**

In the present survey, we investigate the eligible studies for the sensitivity and specificity of rapid tests and explore the factors that influence the result to help better diagnose COVID-19 infection. 20 studies met the inclusion criteria which imposed 33 different tests.

**Results:**

Our findings showed the type of sample, the type of assay, the time of sampling, and the load of virus influence on the sensitivity of RDTs.

**Conclusion:**

This research extends our knowledge of how to improve the sensitivity of RDTs to better diagnose the infected patients to address the controlling COVID-19 pandemic.

**Supplementary Information:**

The online version contains supplementary material available at 10.1186/s13000-022-01215-6.

## Introduction

Early coronavirus infection 2019 (COVID-19) detection is an important and challenging issue to prevent its rapid spread. Real-time PCR (RT-PCR) is a standard gold test used to detect COVID-19 in the laboratory. Despite the high sensitivity of RT-PCR, it was reported that 20–67% of infected patients have false-negative results, but RT-PCR cannot differentiate between infectious and non-infectious SARS-CoV-2 particles [1, 2]. Rapid diagnostic tests (RDTs) for SARS-CoV-2 detection in pharyngeal and blood specimens are routinely employed as point-of-care testing. Limited time and expense, as well as the need of specialized training, make these approaches more appealing, but their major limitation is their low sensitivity. It was shown that some infected individuals still have a positive RT-PCR after final recovery. This false positive can be the cause of existing RNA particles [3], while this has not been reported for RDTs. To detect the IgG and IgM Abs in the blood, plasma, and serum of patients with COVID-19, in addition to the rapid antigen (Ag) test, the rapid antibody (Ab) test is considered a timely point-of-care test [4, 5]. In a recent study, Ricks et al., comparing the two methods of RDTs and RT-PCR due to the health system cost and health impact, stated that despite the low sensitivity of RDTs to RT-PCR, it has valuable to identify the patients infected with reduced costs [6]. Besides, the ability to perform RDTs during the night, weekends, and holidays has made it a significant advantage for diagnosis in emergency medical departments.

Extensive studies have examined the usability of RDTs based on their sensitivity and specificity compared to RT-PCR. However, the reported inconsistent findings need more investigation in order to arrive at a meaningful conclusion. Several studies have shown that fast blood and throat samples had comparable sensitivities to RT-PCR, while others have found lower sensitivity, particularly in asymptomatic individuals [7–10]. These controversial results may be due to different samples and testing to various stages of infection, as many COVID-19 antigen tests are rapidly evolving and need to be systematically indicated in all of these available tests. Recent systematic reviews and Meta-analyzes of the literature have focused on the accuracy of point-of-care tests and diagnostic tests, but no detailed studies were performed on the accuracy of Ag-RDTs in the diagnosis of COVID-19 [11, 12].

In the present systematic review, we attempt to assess the diagnostic utility of antigen detection rapid diagnostic tests for COVID-19 versus RT-PCR in a different types of samples and different stages of infection determine the usability of rapid tests in the best time and sample and explore the factors that influence the result to help better diagnose COVID-19 infection.

## Materials and methods

This review was performed following the PRISMA (Preferred Reporting Items for Systematic reviews and Meta-Analyses) and MOOSE (Meta-analyses Of Observational Studies in Epidemiology) guidelines [13, 14].

### Search strategy

To evaluate the usability of rapid tests compared with RT-PCR, we systematically searched the electronic database, including Scopus, Medline/PubMed, EMBASE, Web of sciences (WOS), and Cochrane library using Mesh-standardized keywords: (((Rapid antigen detection test * OR RDT*) OR “Rapid Antigen Test ”[Mesh] OR “point of care testing”) AND (“Real-time PCR”[Mesh] OR “RT-PCR” OR ‎“Molecular diagnostic test” OR “RNA virus”) AND (“2019 nCoV” OR 2019nCoV OR “2019 novel coronavirus” OR “COVID 19” OR COVID19 OR “new coronavirus” OR “novel coronavirus” OR “novel coronavirus” OR “SARS CoV-2” OR (Wuhan AND (coronavirus OR “corona virus”) OR “COVID-19” OR “severe acute respiratory syndrome coronavirus 2”)) until Jan 2021. There is no restriction for time and language, and the citation lists of selected articles were hand-searched for additional papers.

### Data extraction

Two reviewers independently screened the titles and abstracts of all initially found articles. Information was extracted from selected studies including the name of author, country, sample size, mean age, rapid diagnostic kits, true positive, false positive, false negative, true negative results, sensitivity, and specificity. A third reviewer was consulted to resolve any disagreements between reviewers by discussion until consensus was reached.

### Eligible criteria

To understand the sensitivity and specificity of RDTs, studies that evaluated these parameters were selected. Inclusion criteria were considered as follows: the evaluation of the sensitivity and specificity of RDTs compared to the RT-PCR. All types of studies, including case/control, cohort, cross-sectional, and clinical trial studies, were included. Studies that evaluated seroprevalence, studies that investigated just cell culture assay, case reports, reviews, and studies reporting cases with incomplete information were excluded.

### Statistical analysis

Cochran Chi-square test and *I*^*2*^ were used to assess the heterogeneity among studies. A fixed-effects model was used when *I2* < 50%, while a random-effects model was selected in the case of *I2* > 50%. Fixed- effect model assumes that the population effect sizes are the same for all studies [15]. In contrast, the random-effects model attempted to generalize findings beyond the included studies by assuming that the selected studies are random samples from a larger population [16]. To compare the sensitivity and specificity in RDTs compared with the RT-PCR, 95% confidence intervals (CI) were used. According to the heterogeneity test results, either Der Simonian’s and Laird’s random‑effects method or Mantel‑Haenszel’s fixed‑effects method were used to estimate the overall sensitivity and specificity and 95% confidence intervals [17]. Moreover, subgroup analysis was implemented based on the type of specimen (nasopharyngeal swab, throat washing and bronchoalveolar fluids, and Nasal sample), and symptomatic or asymptomatic patinates as an important variable which may cause heterogeneity between different samples or influence of onset of symptoms. The Egger’s test was used to investigate small study effects due to potential publication bias [18, 19]. If the findings were statistically heterogeneous, a sensitivity analysis was performed to establish the cause of heterogeneity. The randomized effects model was utilized for meta-analysis after considerable clinical heterogeneity was eliminated. *P* < 0.05 was considered the statistical significance (2-sided). All data were analyzed using STAT 16 (STATA Corporation, College Station, Texas).

## Result

**Fig. 1:**
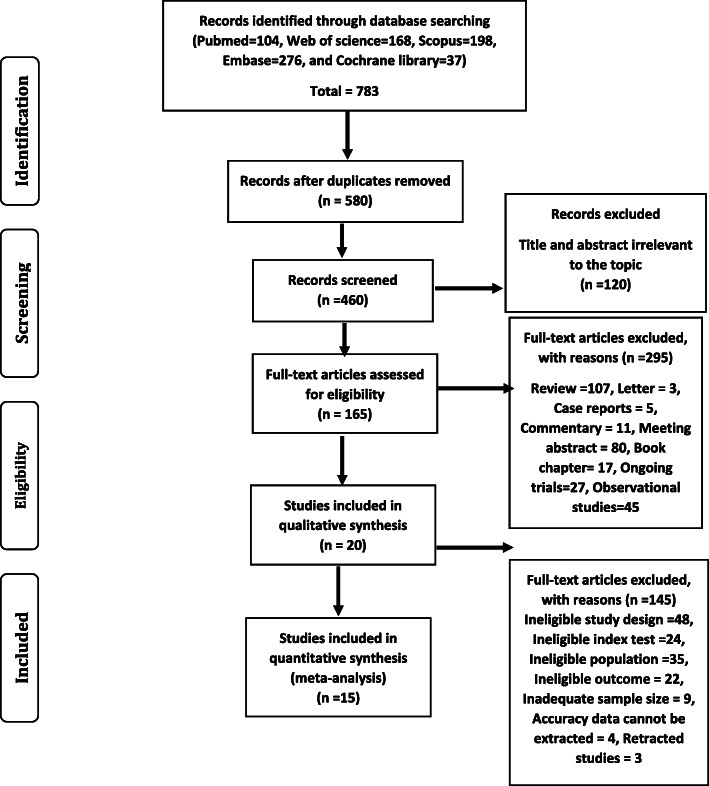
Flow diagram of the study selection process

In general, 783 studies were initially collected (Fig. 1). Finally, 20 studies met our inclusion criteria, which imposed 33 different tests (including 26,056 patients, mean age range from 20.5 to 53.14 years). Eleven studies (55%) evaluated nasopharyngeal swabs [5, 6, 8, 11–19]. Three included studies investigated various types of samples with just one assay [20–22], whereas the other two articles examined one type of sample with different assays to compare the sensitivity and specificity [8, 9]. A single study performed a similar series of experiments but for rapid Abs tests with finger-stick whole-blood [10], whereas other two studies were used the same rapid Abs tests from patient’s serum [23, 24]. Characteristics of included studies are shown in Table 1.

**Table 1:** Characteristics of included studies

Study ID	Country	Rapid Kit	Mean Age (year)	No. of Patients	Source
Agulló et al.	Spain	Panbio COVID-19 Ag-RDT	36.7	659	NP, Nasal, Saliva, Nasal + saliva samples
Abdelrazik et al.	Egypt	BIOCREDIT COVID-19 Ag kit	NR	310	NP swab
Albert et al.	Spain	Panbio™COVID-19 Ag	20.5	412	NP swab
Ciotti et al.	Italy	COVID-19 Ag Respi‐Strip (Coris BioConcept)	53.14	50	NP swab
Kohmer et al.	Germany	R-Biopharm, Roche, NADAL, LumiraDx	NR	100	NP swab
Linares et al.	Spain	Abbott Rapid Diagnostic Jena GmbH	NR	255	NP swab
Nalumansia et al.	Uganda	STANDARD Q COVID-19 Ag	34	262	NP swab
Pilarowski et al.	USA	Abbott BinaxNOWTM COVID-19 Ag	NR	217	anterior swab
Salvagno et al.	Italy	Roche	46	321	NP
Schildgen et al.	Germany	RapiGEN (Gyeonggi-do, Korea), Abbott (Cologne, Germany), Roche	NR	73	throat washing and bronchoalveolar fluids
Scohy et al.	Belgium	COVID-19 Ag Respi-Strip (Coris Bioconcept)	52.25	148	NP
Toptan et al.	Germany	R-Biopharm	NR	70	NP
Torres et al.	Spain		42.5	634	NP
CK Mak et al.	China	BIOCREDIT COVID-19 Ag	NR	35	NP aspirate and throat swab, NP and throat swab, sputum, throat saliva
Prince-Guerra et al.	USA	BinaxNOW antigen	46.75	3419	Anterior nasal swabs

Abbreviations: *NP* nasopharyngeal, *NR* not reported

**Fig. 2:**
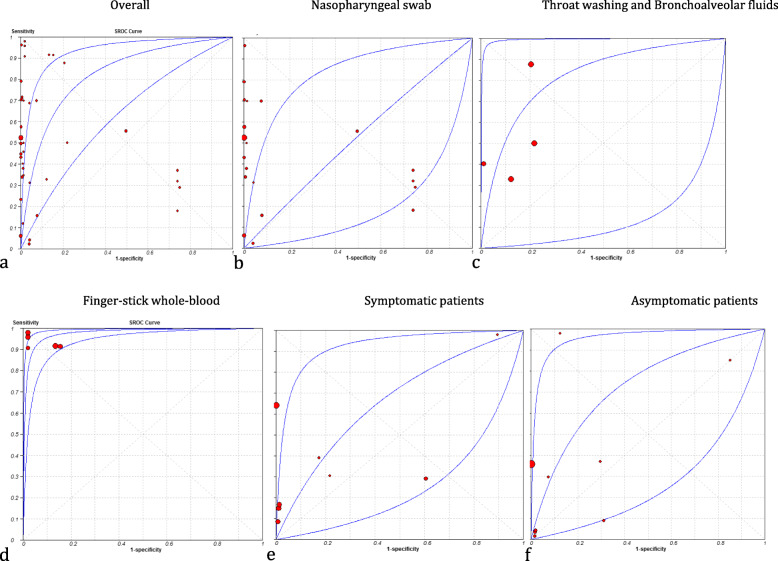
Summary receiver operating characteristic curve (ROC curve). Estimates of sensitivity and specificity for each study are

Our pooled analysis has revealed that the sensitivity and specificity of RDTs were … and…, respectively. Some literatures analyzed the different types of specimens [25–27], some analyzed the sensitivity and specificity during different stages of infection [9, 25, 26, 28, 29], and some compared the diagnostic accuracy of RDTs with RT-PCR based viral load of viruses [8, 20, 28, 30–33]. The characteristics of the diagnostic values of included studies are shown in Table 2. The results of Cochrane Q and *I*^*2*^ statistics showed significant heterogeneity in sensitivity and specificity, so estimations of sensitivity and specificity were obtained using a random effect model. In further analysis, data were analyzed based on the type of sample and symptomatic or asymptomatic patients Table S1, S2, S3, S4, S5 and Figure S1, S2, S3, S4, S5. Summary ROC curves constructed for all assays based on Monte Carlo simulations are shown in Fig. 2. It did not apply to assessing the false results, because 7 studies (35%) did not focus on false results [8, 9, 20, 22, 24, 26, 34, 35]. Some of the current kinds of literature which focused on Ab rapid tests were excluded due to reporting the results as the separate sensitivity for IgG and IgM or analyzed data based on onset of symptoms [4, 36].

**Table 2:** Pooled analysis of sensitivity and specificity of included studies with 95% confidence interval

Study ID	Sensitivity	Specificity	Positive LR	Negative LR	DOR
Agullo et al.	0.576 (0.487–0.661)	0.998 (0.989-1.000)	299.39 (42.023–2133.1)	0.425 (0.348–0.519)	704.36 (96.091–5163)
Agullo et al.	0.447 (0.360–0.536)	1.000 (0.993-1.000)	472.42 (29.398–7591.7)	0.553 (0.475–0.645)	854.05 (52.241-13962.1)
Agullo et al.	0.231 (0.160–0.317)	1.000 (0.992-1.000)	228.93 (14.076–3723.5)	0.767 (0.696–0.846)	298.41 (18.060-4930.7)
Agullo et al.	0.496 (0.404–0.588)	1.000 (0.992-1.000)	485.98 (30.265–7803.7)	0.505 (0.423–0.602)	963.08 (58.809-15771.9)
Abdelrazik et al.	0.431 (0.359–0.505)	1.000 (0.989-1.000)	286.33 (17.860-4590.3)	0.570 (0.503–0.645)	502.65 (30.910-8173.8)
Albert et al.	0.796 (0.665–0.894)	1.000 (0.990-1.000)	567.87 (35.470-9091.7)	0.209 (0.125–0.350)	2712.1 (157.06-46833.5)
Ciotti et al.	0.308 (0.170–0.476)	1.000 (0.715-1.000)	7.500 (0.478–117.57)	0.717 (0.564–0.912)	10.455 (0.570-191.78)
Kohmer et al.	0.290 (0.204–0.389)	0.250 (0.169–0.347)	0.387 (0.279–0.536)	2.840 (1.978–4.078)	0.136 (0.073–0.255)
Kohmer et al.	0.320 (0.230–0.421)	0.260 (0.177–0.357)	0.432(0.318–0.589)	2.615 (1.830–3.737)	0.165 (0.090–0.305)
Kohmer et al.	0.180 (0.110–0.269)	0.260 (0.177–0.357)	0.243 (0.158–0.375)	3.154 (2.238–4.445)	0.077 (0.039–0.152)
Kohmer et al.	0.370 (0.276–0.472)	0.260 (0.177–0.357)	0.500 (0.378–0.662)	2.423 (1.685–3.484)	0.206 (0.113–0.377)
Linares et al.	0.157 (0.114–0.207)	0.922 (0.881–0.951)	2.000 (1.203–3.324)	0.915 (0.858–0.975)	2.186 (1.239–3.857)
Nalumansia et al.	0.700 (0.594–0.792)	0.924 (0.874–0.959)	9.262 (5.398–15.891)	0.325 (0.236–0.446)	28.538 (13.848–58.814)
Pilarowski et al.	0.023 (0.008–0.053)	0.960 (0.925–0.982)	0.576 (0.196–1.691)	1.018 (0.984–1.052)	0.566 (0.187–1.717)
Pilarowski et al.	0.556 (0.212–0.863)	0.503 (0.462–0.545)	1.119 (0.620–2.018)	0.883 (0.423–1.841)	1.267 (0.337–4.767)
Salvagno et al.	0.340 (0.288–0.394)	0.994 (0.978–0.999)	54.500 (13.575–218.81)	0.665 (0.614–0.719)	82.007 (20.035–335.66)
Schildgen et al.	0.329 (0.223–0.449)	0.877 (0.779–0.942)	2.667 (1.332–5.338)	0.766 (0.638–0.919)	3.483 (1.486–8.162)
Schildgen et al.	0.500 (0.381–0.619)	0.781 (0.669–0.869)	2.281 (1.399–3.721)	0.640 (0.495–0.829)	3.563 (1.738–7.302)
Schildgen et al.	0.877 (0.779–0.942)	0.795 (0.684–0.880)	4.267 (2.696–6.753)	0.155 (0.083–0.289)	27.496 (11.184–67.599)
Scohy et al.	0.387 (0.291–0.472)	1.000 (0.916-1.000)	32.608 (2.053–517.87)	0.628 (0.544–0.725)	51.913 (3.118–864.30)
Toptan et al.	0.500 (0.319–0.681)	1.000 (0.907-1.000)	39.000 (2.432–625.53)	0.506 (0.359–0.714)	77.000 (4.357–1360.8)
Torres et al.	0.060 (0.043–0.081)	1.000 (0.994-1.000)	77.000 (4.741–1250.6)	0.940 (0.922–0.959)	81.905 (5.021–1336.2)
CK Mak et al.	0.343 (0.191–0.522)	1.000 (0.900-1.000)	25.000 (1.538–406.50)	0.662 (0.520–0.843)	37.766 (2.132–668.99)
CK Mak et al.	0.457 (0.288–0.634)	1.000 (0.900-1.000)	33.000 (2.057–529.44)	0.549 (0.406–0.744)	60.077 (3.416–1056.6)
CK Mak et al.	0.111 (0.037–0.241)	1.000 (0.921-1.000)	11.000 (0.626–193.25)	0.890 (0.797–0.994)	12.358 (0.663–230.48)
CK Mak et al.	0.400 (0.257–0.557)	1.000 (0.921-1.000)	37.000 (2.297–595.89)	0.604 (0.476–0.768)	61.218 (3.546–1056.9)
Prince-Guerra et al.	0.525 (0.467–0.583)	0.999 (0.997-1.000)	409.57 (152.91–1097.0)	0.476 (0.422–0.536)	861.29 (314.78-2356.6)
Courtellemont et al.	0.967 (0.918–0.991)	1.000 (0.971-1.000)	246.56 (15.502–3921.4)	0.037 (0.015–0.092)	6658.3 (354.65-125004.7)
Courtellemont et al.	0.706 (0.525–0.849)	1.000 (0.897-1.000)	49.000 (3.100-774.56)	0.304 (0.183–0.506)	161.0 (9.002–2879.3)
Pere et al.	0.958 (0.857–0.995)	0.981 (0.897-1.000)	49.833 (7.147–347.45)	0.042 (0.011–0.165)	1173.0 (102.92-13368.3)
Pere et al.	0.917 (0.800-0.977)	0.865 (0.742–0.944)	6.810 (3.401–13.636)	0.096 (0.037–0.248)	70.714 (19.333–258.66)
Pere et al.	0.923 (0.749–0.991)	1.000 (0.858-1.000)	45.37 (2.910–707.30)	0.094 (0.029–0.308)	480.20 (21.093-10527.9)
Pere et al.	0.979 (0.889–0.999)	0.981 (0.897-1.000)	50.917 (7.306–354.84)	0.021 (0.003–0.148)	2397.0 (145.76–39,418)
Pere et al.	0.915 (0.796–0.976)	0.846 (0.719–0.931)	5.947 (3.125–11.316)	0.101 (0.039–0.259)	59.125 (16.576–210.89)
Fabre et al.	0.041 (0.017–0.083)	0.959 (0.917–0.981)	1.000 (0.359–2.789)	1.000 (0.957–1.045)	1.000 (0.343–2.916)
Cerutti et al.	0.706 (0.612–0.790)	1.000 (0.983-1.000)	312.82 (19.576–4998.8)	0.296 (0.222–0.395)	1056.4 (63.918-17459.1)
Montesinosa et al.	0.719 (0.632–0.795)	1.000 (0.950-1.000)	104.69 (6.597–1661.4)	0.285 (0.216–0.375)	367.47 (22.176–6089.1)
Montesinosa et al.	0.688 (0.600-0.766)	0.958 (0.883–0.991)	16.500 (5.417–50.263)	0.326 (0.251–0.424)	50.600 (15.016–170.51)
Montesinosa et al.	0.711 (0.624–0.788)	1.000 (0.950-1.000)	103.56 (6.525–1643.6)	0.293 (0.223–0.384)	353.80 (21.360-5860.2)

## Discussion

Reverse transcription-quantitative PCR (RT-qPCR) is widely used to diagnose COVID-19. However, these tests cannot be performed in local clinics where RT-qPCR testing is unavailable, so rapid antigen tests (RATs) for COVID-19 are used for rapid diagnosis [37]. Therefore, samples should be moved to locations that have RT-qPCR capability. This process delays the test result and increases the anxiety of patients suspected of having COVID-19. To solve this problem, RATs, which do not require special and expensive machines, have been approved to detect COVID-19, and the sensitivity of these tests was compared with RT-qPCR methods [38–40].

The advent of rapid antigen diagnostic tests for SARS-CoV-2 has posed many challenges in terms of accessibility and performance. Further evidence on how to conduct and use such tests is needed to reach a definitive conclusion about using them. Choosing the proper virological test for COVID-19 affects early detection, rapid control, and prophylactic responses to virus outbreaks. Early detection allows promptly removing infections, thus minimizing transmission opportunities [1]. Recent interpretations have highlighted the potential application of high-frequency, low-sensitivity experiments in asymptomatic individuals. Current analysis suggests a less sensitive, but more accessible test may be preferable to detect symptomatic COVID- 19 [41]. COVID-19 Ag-RTD is a recent generation sensitive and specific antigen test for the qualitative detection of SARS-CoV-2 antigen in human nasopharyngeal swab samples. Ag-RDTs may be based on more accessible and uncomplicated samples that can even be self-collected, such as nasal swab or saliva, in addition to the nasopharyngeal swab, which needs qualified healthcare personnel and personal protective equipment to collect. Ag-RDTs directly detect SARS-CoV-2 proteins produced by virus replication in respiratory secretions [25]. Ag-RDTs, compared to methods such as nucleic acid amplification tests (NAAT) and RT-PCR, are relatively inexpensive, easy to perform, do not require infrastructure, and allow care results to be achieved in minutes. Assessments by Ricks et al. strongly supported Ag-RDT for evaluating symptomatic individuals, making it less cost-effective and practical than relying on NAT and clinical judgment [6]. They announced Ag-RDTs are more available than other methods but are usually less sensitive and specific. However, RDTs read samples with high levels of virus as positive, and even the most sensitive RDTs read samples with small amounts of the virus as negative. Ag-RDTs are estimated to be less than 80% sensitive to COVID-19, compared to > 90% for NAT [42]. Despite the lower sensitivity of Ag-RDT, it is more helpful in guiding patient management at point-of-care, repeat testing, and large-scale public health decisions on time to prevent transmission [31]. Yamayoshi et al. determined that soaking the sample immediately in the lease buffer might boost the sensitivity of RDTs after examining different samples [37]. RDTs are less sensitive for nasal examination swabs and saliva samples, according to studies examining various kinds of specimens [25]. Performing RDT with different assays is an important issue which can cause various sensitivities. A study to determine the sensitivity of varying RDT assays showed different results for positive controls in various assays that could provide appropriate methods to identify the distinct Ags and consequently test accuracy [26].

Our results showed that most of the collected samples are nasopharyngeal swabs, the study of which increases the sensitivity of RDTs results. RT-PCR analyses showed higher sensitivity in infected patients with lower Ct values. In principle, RDT susceptibility is reduced in asymptomatic patients or patients with a lower viral load. Cerutti et al., using the amount of Ct by RT-PCR, concluded that in R-Ag positive samples, Ct was significantly lower than in R-Ag negative samples. Most RT-PCR positive and R-Ag negative samples reported negative results during cell culture, which may explain the higher R-Ag sensitivity [43]. Asymptomatic persons, on the other hand, should not be ruled out, according to Pilarowski et al., since asymptomatic patients may also have a high viral load [27]. Albert et al. evaluated cell culture sensitivity with RT-PCR to identify COVID-19 in addition to RDTs. The results showed that susceptibility was reduced in cell cultures with a lower viral load, such as RDTs [31].

To assess the sensitivity of RDTs, Eshghifar et al. used different concentrations of heat-inactivated COVID-19 virus to evaluate the cut-off detection for RDTs. Their results showed that they could not determine a cut-off and reported RDTs positively only in patients with high viral load [22]. It’s impossible to rule out the possibility of interactions throughout the test. According to certain studies, testing fewer than 5 days after the beginning of symptoms improves sensitivity [9, 22, 34], which contradicts prior claims that the infectious virus load declines after 7 to 10 days [44].Depending on the age of participants, Albert et al. reported that RDTs were less sensitive in children than in adults [31].

Our analysis showed that rapid detection of Ab is less sensitive to RDT and is associated with many false positives. In their study, Pere et al. found that a recent history of infection with cold Abs led to a false positive increase in rapid Ab testing, especially for IgM, which reduced the validity of the test [10]. Faber et al. carried out a similar investigation, and their results revealed that false positives are more likely among pregnant women [23]. One of the causes for the quick Ab test’s poor accuracy might be the patients’ short-term safety [45]. Many studies focus on the accuracy of serological tests, and as expected, their findings show less accuracy in serological tests [11, 46]. Our findings showed the type of sample, type of assay, time of sampling, and the load of virus influence on the sensitivity of RDTs. Despite domestic vaccination, there are still significant new cases, especially in low- and middle-income countries. Hence, advances in rapid detection at lower costs remain a significant challenge. The findings of this meta-analysis have important implications for the development of the RDT technique in the detection of SARS-CoV-2 with the highest possible sensitivity and specificity.

## Conclusions

The main goal of the current study was to determine the accuracy of RDTs. We showed the explanations for low sensitivity in RDTs such as type of specimen, the timing of sampling, type of assay, and viral load.; and by considering them, RDTs can be used to identify the suspected patients in the early stage of disease with desirable sensitivity and specificity and help control COVID-19 pandemic.

## Limitations

The major limitation of this study is the accuracy of RDTs affecting the spread of the COVID-19 virus or not? In this regard, more research is required to determine the efficacy of RDTs in detecting the various type of COVID-19 viruses. Another issue that was not addressed in this study was whether false results in RDTs. This is because seven studies did not report false results.

## Supplementary information

**Additional file 1.**

**Additional file 2.**

**Additional file 3.**

## Data Availability

The datasets generated and/or analyzed during the current study are available in the [Pubmed, Web of Science, Scopus, EM Base] repository.

## Abbreviations

COVID-19: Coronavirus infection 2019
RT-PCR: Real-time PCR
Ag: Antigen
Ab: Antibody
WOS: Web of sciences
CI: Confidence intervals
RT-qPCR: Reverse transcription-quantitative PCR
RATs: Rapid antigen tests
NAAT: Nucleic acid amplification tests
