# Supplementary material for: Diagnostic utility of antigen detection rapid diagnostic tests for Covid-19: a systematic review and meta-analysis
Source: Diagn Pathol. 2022 Apr 13;17:36. doi: 10.1186/s13000-022-01215-6 (PMC9005339; doi:10.1186/s13000-022-01215-6)
Supplement: Supplementary file 2 — Additional file 2. [file 13000_2022_1215_MOESM2_ESM.pdf]

ENGLISH EDiT WWW.NativeEnglishEdit.com

EDITORIAL CERTIFICATE LETTER

This document is to certify that the manuscript listed below was edited for proper English language, grammar, punctuation, spelling, and overall style by one of the highly qualified subject-expert native English speaking editors at **NativeEnglishEdit.com**

The substantive content of the article mentioned below remains the full responsibility of the author/authors:

TITLE OF ARTICLE:
DIAGNOSTIC UTILITY OF ANTIGEN DETECTION RAPID DIAGNOSTIC TESTS FOR COVID- 19: A SYSTEMATIC REVIEW AND META-ANALYSIS

AUTHOR(S):

SOMAYEH GHASEMI, NARGES NAZARI HARMOOSHI, FAKHER RAHIM

REFER CODE:
250H EE-2022-333318335-MINA.EBRAHIMI271-2613 MED

Native English Edit
www.NativeEnglishEdit.com

Documents receiving this certification should be English-ready for publication; however, the author has the ability to accept or reject our suggestions and changes.
This certificate may be verified at:
www.NativeEnglishEdit.com
London
East End Road 27, N 3 3QT
United Kingdom
