# Supplementary material for: Diagnostic utility of antigen detection rapid diagnostic tests for Covid-19: a systematic review and meta-analysis
Source: Diagn Pathol. 2022 Apr 13;17:36. doi: 10.1186/s13000-022-01215-6 (PMC9005339; doi:10.1186/s13000-022-01215-6)
Supplement: Supplementary file 3 — Additional file 3. [file 13000_2022_1215_MOESM3_ESM.docx]

**Supplementary**

**Diagnostic Utility of Antigen Detection Rapid Diagnostic Tests for Covid- 19: A
Systematic Review and Meta-Analysis**

**Somaye Ghasemi 1, Narges Nazari Harmooshi2,3, Fakher Rahim4***

| Study | Sensitivity | Specificity | Positive LR | Negative LR | DOR |
| --- | --- | --- | --- | --- | --- |
| Agullo et al. (27) | 0.576 (0.487- 0.661) | 0.998 (0.989 - 1.000) | 299.39 (42.023 -2133.1) | 0.425 (0.348 - 0.519) | 704.36 (96.091 - 5163.0) |
| Abdelrazik et al. (37) | 0.431 (0.359- 0.505) | 1.000 (0.989 - 1.000) | 286.33 (17.860 -4590.3) | 0.570 (0.503 - 0.645) | 502.65(30.910 - 8173.8) |
| Albert et al. (21) | 0.796 (0.665- 0.894) | 1.000 (0.990 - 1.000) | 567.87 (35.470 -9091.7) | 0.209 (0.125 - 0.350) | 2712.1 (157.06 - 46833.5) |
| Ciotti et al.(6) | 0.308 (0.170- 0.476) | 1.000 (0.715 - 1.000) | 7.500 (0.478 -117.57) | 0.717 (0.564 - 0.912) | 10.455 (0.570 - 191.78) |
| Kohmer et al.(7) | 0.290 (0.204 - 0.389) | 0.250 (0.169 - 0.347) | 0.387 (0.279 -0.536) | 2.840 (1.978 - 4.078) | 0.136 (0.073 - 0.255) |
| Kohmer et al.(7) | 0.320 (0.230 - 0.421) | 0.260 (0.177 - 0.357) | 0.432 (0.318 -0.589) | 2.615 (1.830 - 3.737) | 0.165 (0.090 - 0.305) |
| Kohmer et al.(7) | 0.180 (0.110 - 0.269) | 0.260 (0.177 - 0.357) | 0.243 (0.158 -0.375) | 3.154 (2.238 - 4.445) | 0.077 (0.039 - 0.152) |
| Kohmer et al.(7) | 0.370 (0.276 - 0.472) | 0.260 (0.177 - 0.357) | 0.500 (0.378 -0.662) | 2.423 (1.685 - 3.484) | 0.206 (0.113 - 0.377) |
| Linares et al. (22) | 0.157 (0.114 - 0.207) | 0.922 (0.881 - 0.951) | 2.000 (1.203 -3.324) | 0.915 (0.858 - 0.975) | 2.186 (1.239- 3.857) |
| Nalumansia et al.(23) | 0.700 (0.594 - 0.792) | 0.924 (0.874 - 0.959) | 9.262 (5.398 -15.891) | 0.325 (0.236 - 0.446) | 28.538 (13.848 - 58.814) |
| Pilarowski et al.(34) | 0.023 (0.008 - 0.053) | 0.960 (0.925 - 0.982) | 0.576 (0.196 -1.691) | 1.018 (0.984 - 1.052) | 0.566 (0.187 - 1.717) |
| Pilarowski et al.(34) | 0.556 (0.212- 0.863) | 0.503 (0.462 - 0.545) | 1.119 (0.620 -2.018) | 0.883 (0.423 - 1.841) | 1.267 (0.337 - 4.767) |
| Salvagno et al. (31) | 0.340 (0.288-0.394) | 0.994 (0.978 - 0.999) | 54.500 (13.575 -218.81) | 0.665 (0.614 - 0.719) | 82.007(20.035- 335.66) |
| Scohy et al. (24) | 0.378 (0.291-0.472) | 1.000 (0.916 - 1.000) | 32.608 (2.053 -517.87) | 0.628 (0.544 - 0.725) | 51.913 (3.118- 864.30) |
| Toptan et al.(25) | 0.500 (0.319 -0.681) | 1.000 (0.907 - 1.000) | 39.000 (2.432 -625.53) | 0.506 (0.359 - 0.714) | 77.000 (4.357 - 1360.8) |
| Torres et al.(26) | 0.060 (0.043-0.081) | 1.000 (0.994 - 1.000) | 77.000 (4.741 -1250.6) | 0.940 (0.922 - 0.959) | 81.905 (5.021- 1336.2) |
| Prince-Guerra et al.(35) | 0.525 (0.467-0.583) | 0.999 (0.997 - 1.000) | 409.57 (152.91 -1097.0) | 0.476 (0.422 - 0.536) | 861.29 (314.78 - 2356.6) |
| Courtellemont et al. (9) | 0.967 (0.918-0.991) | 1.000 (0.971 - 1.000) | 246.56 (15.502 -3921.4) | 0.037 (0.015 - 0.092) | 6658.3 (354.65 - 125004.7) |
| Courtellemont et al. (9) | 0.706 (0.525-0.849) | 1.000 (0.897 - 1.000) | 49.000 (3.100 -774.56) | 0.304 (0.183 - 0.506) | 161.00 (9.002 - 2879.3) |
| Cerutti et al. (36) | 0.706 (0.612- 0.790) | 1.000 (0.983 - 1.000) | 312.82(19.576 - 4998.8) | 0.296 (0.222 - 0.395) | 1056.4 (63.918 - 17459.1) |

**Table S1.** Sub analysis of sensitivity and specificity for nasopharyngeal swab with 95% confidence interval.

**Figure S1.** A forest plot showing the estimates for sensitivity (A) and specificity (B) for nasopharyngeal swab.


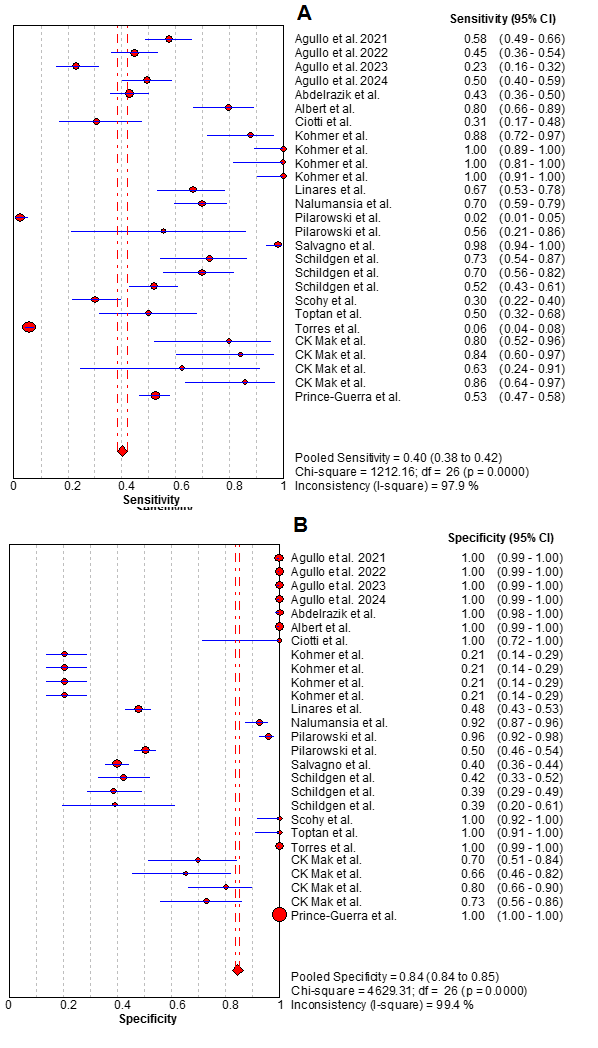


**Table S2.** Sub analysis of sensitivity and specificity for throat washing and broncho-alveolar fluids with 95% confidence interval.

| Study | Sensitivity | Specificity | Positive LR | Negative LR | DOR |
| --- | --- | --- | --- | --- | --- |
| Schildgen et al. (32) | 0.329 (0.223- 0.449) | 0.877 (0.779- 0.942) | 2.667 (1.332 - 5.338) | 0.766 (0.638 - 0.919) | 3.483(1.486- 8.162) |
| Schildgen et al. (32) | 0.500 (0.381- 0.619) | 0.781 (0.669- 0.869) | 2.281 (1.399 - 3.721) | 0.640 (0.495- 0.829) | 3.563 (1.738 - 7.302) |
| Schildgen et al. (32) | 0.877 (0.779- 0.942) | 0.795 (0.684- 0.880) | 4.267 (2.696- 6.753) | 0.155(0.083 - 0.289) | 27.496(11.184- 67.599) |
| CK Mak et al. (18) | 0.400 (0.257- 0.557) | 1.000 (0.921- 1.000) | 37.000 (2.297 - 595.89) | 0.604 (0.476- 0.768) | 61.218 (3.546- 1056.9) |

A

B


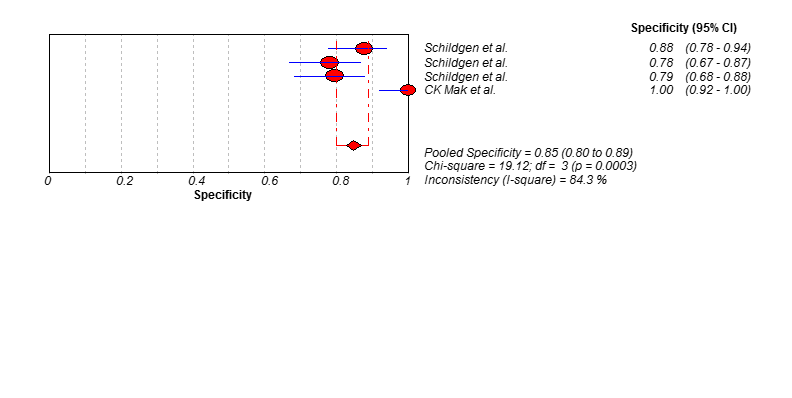


**Figure S2.** A forest plot showing the estimates for sensitivity (A) and specificity (B) for Throat washing and Bronchoalveolar fluids.

**Table S3.** Sub analysis of sensitivity and specificity for finger-stick whole-blood with 95% confidence interval.

| Study | Sensitivity | Specificity | Positive LR | Negative LR | DOR |
| --- | --- | --- | --- | --- | --- |
| Pere et al. (10) | 0.958 (0.857- 0.995) | 0.981 (0.897 - 1.000) | 49.833 (7.147- 347.45) | 0.042 (0.011- 0.165) | 1173.0 (102.92- 13368.3) |
| Pere et al. (10) | 0.917 (0.800- 0.977) | 0.865 (0.742 - 0.944) | 6.810 (3.401 - 13.636) | 0.096 (0.037- 0.248) | 70.714 (19.333- 258.66) |
| Pere et al. (10) | 0.923 (0.749- 0.991) | 1.000 (0.858 - 1.000) | 45.370 (2.910- 707.30) | 0.094 (0.029- 0.308) | 480.20 (21.903- 10527.9) |
| Pere et al. (10) | 0.979 (0.889- 0.999) | 0.981 (0.897- 1.000) | 50.917 (7.306 - 354.84) | 0.021 (0.003- 0.148) | 2397.0 (145.76- 39418.0) |
| Pere et al. (10) | 0.915 (0.796- 0.976) | 0.846 (0.719 - 0.931) | 5.947 (3.125 - 11.316) | 0.101 (0.039- 0.259) | 59.125 (16.576- 210.89) |

A B

ab

**Figure S3.** A forest plot showing the estimates for sensitivity (A) and specificity (B) for Finger-stick whole-blood.

**Table S4.** Sub analysis of sensitivity and specificity for Symptomatic patients with 95% confidence interval.

| Study | Sensitivity | Specificity | Positive LR | Negative LR | DOR |
| --- | --- | --- | --- | --- | --- |
| Agullo et al. (27) | 0.147 (0.111- 0.188) | 1.000 (0.923- 1.000) | 13.871 (0.870 - 221.04) | 0.862 (0.817- 0.908) | 16.099 (0.977- 265.36) |
| Agullo et al. (27) | 0.083 (0.054- 0.120) | 1.000 (0.944- 1.000) | 10.941 (0.675- 177.41) | 0.923 (0.886- 0.961) | 11.854 (0.712- 197.28) |
| Agullo et al. (27) | 0.166 (0.127- 0.210) | 1.000 (0.905- 1.000) | 12.667 (0.799 - 200.93) | 0.844 (0.795- 0.897) | 15.000 (0.908- 247.93) |
| Prince-Guerra et al. (35) | 0.642 (0.566- 0.713) | 1.000 (0.994- 1.000) | 836.18 (52.243- 13383.6) | 0.359 (0.295 - 0.437) | 2329.0 (143.07- 37912.1) |
| Scohy et al. (24) | 0.291 (0.198 - 0.399) | 0.395 (0.292- 0.507) | 0.481 (0.332- 0.697) | 1.794 (1.337- 2.408) | 0.268 (0.142- 0.506) |
| Schildgen et al. (32) | 0.304 (0.132- 0.529) | 0.783 (0.563- 0.925) | 1.400 (0.519- 3.773) | 0.889 (0.629- 1.256) | 1.575 (0.416 - 5.959) |
| Schildgen et al. (32) | 0.391 (0.197- 0.615) | 0.826 (0.612- 0.950) | 2.250 (0.806- 6.279) | 0.737 (0.505- 1.075) | 3.054 (0.780 - 11.959) |
| Schildgen et al. (32) | 1.000 (0.852- 1.000) | 0.087 (0.011- 0.280) | 1.093 (0.942 - 1.268) | 0.200 (0.010- 3.950) | 5.465 (0.248 - 120.37) |

**Figure S4.** A forest plot showing the estimates for sensitivity (A) and specificity (B) for Symptomatic patients.

A

B

**Table S5.** Sub analysis of sensitivity and specificity for asymptomatic patients with 95% confidence interval.

| Study | Sensitivity | Specificity | Positive LR | Negative LR | DOR |
| --- | --- | --- | --- | --- | --- |
| Agullo et al. (27) | 0.034 (0.015- 0.065) | 1.000 (0.872 - 1.000) | 1.992 (0.118-33.587) | 0.982 (0.929- 1.038) | 2.028 (0.114 -36.111) |
| Agullo et al. (27) | 0.014 (0.003- 0.040) | 1.000 (0.881 - 1.000) | 0.972 (0.051-18.363) | 1.000 (0.952- 1.051) | 0.972 (0.049-19.288) |
| Agullo et al. (27) | 0.041 (0.019- 0.076) | 1.000 (0.858 - 1.000) | 2.140 (0.128-35.668) | 0.977 (0.918- 1.040) | 2.191 (0.124-38.806) |
| Prince-Guerra et al. (35) | 0.358 (0.273- 0.449) | 0.998 (0.996 - 1.000) | 220.80 (80.629-604.68) | 0.643 (0.564- 0.734) | 343.23 (120.37-978.66) |
| Scohy et al. (24) | 0.089 (0.025- 0.212) | 0.689 (0.534 - 0.818) | 0.286 (0.102-0.802) | 1.323 (1.065- 1.642) | 0.216 (0.065-0.721) |
| Courtellemont et al. (9) | 1.000 (0.858- 1.000) | 0.886 (0.733 - 0.968) | 7.840 (3.297-18.640) | 0.023 (0.001- 0.356) | 343.00 (17.613-6679.5) |
| Schildgen et al. (32) | 0.296 (0.138- 0.502) | 0.926 (0.757- 0.991) | 4.000 (0.934-17.134) | 0.760 (0.582- 0.993) | 5.263 (1.000-27.690) |
| Schildgen et al. (32) | 0.370 (0.194- 0.576) | 0.704 (0.498- 0.862) | 1.250 (0.584-2.677) | 0.895 (0.613 - 1.307) | 1.397 (0.448- 4.355) |
| Schildgen et al. (32) | 0.852 (0.663 - 0.958) | 0.148 (0.042- 0.337) | 1.000 (0.801-1.249) | 1.000 (0.278 - 3.594) | 1.000 (0.223- 4.489) |

A

B

**Figure S5.** A forest plot showing the estimates for sensitivity (A) and specificity (B) for asymptomatic patients.
