# Supplementary material for: Diagnostic utility of antigen detection rapid diagnostic tests for Covid-19: a systematic review and meta-analysis
Source: Diagn Pathol. 2022 Apr 13;17:36. doi: 10.1186/s13000-022-01215-6 (PMC9005339; doi:10.1186/s13000-022-01215-6)
Supplement: Supplementary file 1 — Additional file 1. [file 13000_2022_1215_MOESM1_ESM.docx]

***Dear referees:***

***We corrected all the raised comments and marked the corrections with track changed to simplify the reviewing process. I appreciate you and your reviewers in advance***

Your manuscript 'Diagnostic Utility of Antigen Detection Rapid Diagnostic Tests for Covid- 19: A Systematic Review and Meta-Analysis' (DPAT-D-21-00568) has been assessed by our reviewers. Although it is of interest, we are unable to consider it for publication in its current form. The reviewers have raised a number of points which we believe would improve the manuscript and may allow a revised version to be published in Diagnostic Pathology.

Reviewer reports:

Reviewer #1: Ghasemi et al. provide an original and eligible study on the evaluation of rapid diagnostic tests for Covid-19. This is an interesting study that will have an obvious impact on improving the diagnosis of COVID-19. The manuscript is generally written well and concisely. However, there are several issues that requires author's attention. Below are some of my comments to improve the article.

Please review and correct the entire text of the manuscript in terms of spelling and English grammar. In some sentences, written and grammatical rules are not observed, for example in the following sentences.

Abstract, line 38: "Despite high sensitivity of RT-PCR, not only it was reported that 20-67% of infected patients have false negative results."

Introduction, line 18: "It was demonstrated that some infected subjects have RT-PCR positive after ultimately recovery, which is not infectious and can be causes of RNA particles (3), whereas it was not reported for RDTs."

Introduction, line 21: "Additional to the rapid antigen (Ag) test, the rapid antibody (Ab) test was considered as a timely point-of-care test to detect IgG and IgM Abs in blood, plasma, and serum of patient with COVID-19 (4,5)."

Introduction, line 26: "In a recent study, Ricks et al. analyzed the health system cost and health impact of using RDTs among hospitalization and mildly symptomatic patient with COVID19, and report that despite the low sensitivity of RDTs compare with RT-PCR, it has avaluable potential in detecting infected patients with reducing costs (6)."

Introduction, line 40: "However, inconsistency in reported results has heightened the need for a comprehensive search to achieving a reliable finding."

Introduction, line 66: "In the present systematic review, we attempt to assess the diagnostic utility of antigen detection rapid diagnostic tests for COVID-19 versus RT-PCR in a different type of samples and different stages of infection determine the usability of rapid tests in best time and sample and explore the factors that influence the result to help better diagnose COVID-19 infection."

And so on…

Also, in some sentences, written and grammatical rules are not observed, for example, in the case of using capital letters, the period after punctuation, parentheses, and commas in the following sentences:

Search Strategy, line 20: " To evaluate the usability of rapid tests compare with RT-PCR, we systematically searched the electronic database, including Scopus, Medline/PubMed, EMBASE, web of sciences (WOS), and Cochrane library using Mesh-standardized keywords: (((Rapid antigen detection test * OR RDT*) OR "Rapid Antigen Test "[Mesh] OR "point of care testing") AND ("Real-time PCR"[Mesh] OR "RT-PCR" OR "Molecular diagnostic test" OR "RNA virus") AND ("2019 nCoV" OR 2019nCoV OR "2019 novel coronavirus" OR "COVID 19" OR COVID19 OR "new coronavirus" OR "novel coronavirus" OR "novel coronavirus" OR "SARS CoV-2" OR (Wuhan AND (coronavirus OR "corona virus") OR "COVID-19" OR "severe acute respiratory syndrome coronavirus 2")) until Jan 2021."

Data extraction, line 45: "Two reviewers (ME (MSc of hematology) and FR (PhD (Ph.D.) of bioinformatic)) independently screened titles and abstracts of all initially found articles."

In addition, in the above sentence, there is no need to mention the name or degree of individuals and basically there is no need to describe the duties of each author in the text of an article.

Eligible Criteria, line 10: "Additionally, letter to editors which reported comprehensive data was included."

The meaning of the above sentence and the purpose for which the letter was sent to "the editor" is unclear and it is not necessary to state with whom you have corresponded.

Statistical Analysis, line 20: "Cochran Chi-square test and I2 were used to assessing heterogeneity among studies."

There is a difference between the "Cochran's Q test" and the "Chi-squared test" (chi-square or χ2 test). Furthermore, it is not clear what is meant by the "I2".

Statistical Analysis, line 20: "Fixed-model assumes that the population effect sizes are the same for all studies (16)."

In the above sentence, it seems that the meaning of "Fixed-model" is actually "Fixed-effects model", in which case it should be corrected.

Answer:

Many thanks for the dear reviewer attention for above comments. In regard with the reviewer request, all manuscript was edited for proper English language, grammar, punctuation, spelling, and overall style by one of the highly qualified subject-expert native English-speaking editors at NativeEnglishEdit.com and the certificate was attached.

***Thanks for your attention and useful comments.***
